# Efficacy, safety, and complications of manta vascular closure device in VA-ECMO decannulation: A systematic review and meta-analysis

**DOI:** 10.1177/11297298251325391

**Published:** 2025-03-21

**Authors:** Joana Nunes-Carvalho, Eduardo Silva, Paolo Spath, Leonardo Araújo-Andrade, Nicola Troisi, João Rocha Neves

**Affiliations:** 1 Faculty of Medicine of the University of Porto, Porto, Portugal; 2 Angiology and Vascular Surgery Department, Coimbra Local Health Unit, Coimbra, Portugal; 3 Vascular Surgery, DIMEC, University of Bologna, Bologna, Italy; 4 Vascular Surgery Unit, Hospital «Infermi», AUSL Romagna, Rimini, Italy; 5 Centro Hospitalar Universitário São João—Unidade Local de Saúde São João, Porto, Portugal; 6 Department of Biomedicine, Unity of Anatomy, Faculty of Medicine of the University of Porto, Porto, Portugal; 7 Vascular Surgery Unit, Department of Translational Research and New Technologies in Medicine and Surgery, University of Pisa, Pisa, Italy; 8 RISE-Health, Departamento de Biomedicina—Unidade de Anatomia, Faculdade de Medicina, Universidade do Porto, Porto, Portugal

**Keywords:** Arterial puncture closure, mechanical circulatory support, closure device, collagen plug, venoarterial extracorporeal membrane oxygenation

## Abstract

**Background:**

VenoArterial (VA)-ExtraCorporeal Membrane Oxygenation (ECMO) decannulation was traditionally performed surgically, often resulting in high rates of periprocedural complications such as surgical site infections, bleeding, and elevated patient mobilization costs. The advent of percutaneous techniques, particularly the MANTA^®^ vascular closure device (MVCD), has significantly reduced these risks by enabling faster and safer decannulation. This study aimed to systematically review the success rates and complications associated with the use of percutaneous closure devices for VA-ECMO decannulation.

**Objective:**

Therefore, this systematic review with meta-analysis aims to evaluate the success rates and complications associated with the use of MVCD device for VA-ECMO decannulation.

**Materials and methods:**

A systematic search was conducted across Pubmed, Web of Science, and Cochrane databases to identify studies evaluating postoperative outcomes in patients undergoing VA-ECMO decannulation using the MANTA^®^ vascular closure device. The MANTA^®^ efficacy, incidence of emergent open repair, arterial thrombosis, acute limb ischemia, pseudoaneurysms, and major bleeding were pooled by fixed-effects meta-analysis, with sources of heterogeneity being explored by meta-regression. Assessment of studies’ quality was performed using the National Heart, Lung, and Blood Institute (NHLBI) Study Quality Assessment Tool for observational cohorts and case-series studies.

**Results:**

Seven observational studies with 235 patients were included in the final analysis. Overall efficacy of MVCD in VA-ECMO decannulation was 94.8% (95% CI 91.8%–97.9%). In 235 patients, the incidence of emergency open repair after MVCD failure was 3.7% (95% CI 1.3%–6.1%), the incidence of arterial thrombosis was 7.1% (95% CI 2.9%–11.3%), the incidence of pseudoaneurysms was 3.2% (95% CI 0.9%–5.5%), the incidence of acute limb ischemia was 5.0% (95% CI 2.3%–7.8%), and the incidence of major arterial bleeding was 4.1% (95% CI 1.6%–6.7%).

**Conclusion:**

This systematic review and meta-analysis highlights the safety and efficacy of the MANTA^®^ vascular closure device in achieving hemostasis following VA-ECMO decannulation, demonstrating an acceptable success rate and a low incidence of major complications. Further studies with larger cohorts are necessary to validate these findings and to address the limitations of this preliminary experience.

## Introduction

The increasing adoption of ExtraCorporeal Membrane Oxygenation (ECMO) as a cardiopulmonary support system has been driven by its relatively straightforward implementation, the growing presence of mobile ECMO teams, and the widening scope of clinical indications for its use.^1^ In fact, data from the Extracorporeal Life Support Organization (ELSO) Registry reported 16,803 ECMO cases in 2022, highlighting its expanding role in patient care.^2^

VA-ECMO can be established using either a central or peripheral configuration. Typically, peripheral VA-ECMO is initiated by placing cannulas in the common femoral vein (CFV) for blood drainage and the common femoral artery (CFA) for the return of oxygenated blood.^3^ Hemostasis for venous access sites can often be achieved through hemostatic sutures or extended manual compression, but repairing large arteriotomy wounds typically requires more complex interventions. While surgical decannulation was the standard practice initially, it often comes with significant risks, including high costs associated with patient mobilization and a considerable rate of complications, such as bleeding, ischemia, compartment syndrome, amputations, and infections in a relevant proportion.^4^ However, advancements in medical technology have made it possible to both implant and decannulate peripheral VA-ECMO using fully percutaneous techniques, eliminating the need for open surgery.^5,6^ A study by Haddad et al. reported infection rates as high as 45%.^7^ In response, percutaneous techniques using suture-mediated closure devices have been increasingly adopted to minimize these complications.^6^

Devices like the MANTA^®^ Vascular Closure Device (MVCD; Teleflex, Wayne, PA, USA) enable clinicians to quickly achieve hemostasis while preserving vessel integrity following the removal of large-bore arterial cannulas used in peripheral VA-ECMO. The MANTA^®^ VCD is the first vascular closure device designed explicitly for large-bore arteriotomy closure, featuring a poly(lactic-co-glycolic acid) intra-arterial toggle and an extravascular bovine collagen plug.^8^

There is considerable evidence supporting the safety and effectiveness of the MVCD in procedures such as percutaneous endovascular aortic repair (EVAR), and transcatheter aortic valve implantation (TAVI).^9,10^ Additionally, recent studies have demonstrated its successful use in managing vascular access following the removal of devices like the Impella left ventricular support (Abiomed, Danvers, MA, USA), which involves arterial sheaths of 8–10 F.^11^ However, its application in ECMO procedures still needs to be widely examined.

This study aimed to systematically review the safety and effectiveness of the MVCD for percutaneous VA-ECMO decannulation.

## Methods

This systematic review was conducted following the guidelines of the Preferred Reporting Items for Systematic Reviews and Meta-Analyses (PRISMA) Statement and the Assessing the Methodological Quality of Systematic Reviews (AMSTAR) tool.^12,13^ The quality of evidence was assessed using the GRADE (Grading of Recommendations, Assessment, Development, and Evaluations) framework.^14^ Ethical approval from an institutional review board was not required due to the nature of the study. The review protocol has been registered on PROSPERO (reference: CRD42024520215).

The PICO framework for the study can be outlined as follows: Population (P): Patients undergoing percutaneous Veno-Arterial Extracorporeal Membrane Oxygenation (VA-ECMO) decannulation. Intervention (I): Use of the MVCD for vascular access site closure after VA-ECMO. Comparison (C): The MVCD will be evaluated without a direct comparison due to the lack of head-to-head comparisons. Outcome (O): The safety and effectiveness of using the MVCD for percutaneous VA-ECMO decannulation.

### Search strategy

A systematic search was conducted in three databases—PubMed, Web of Science, and Cochrane—on January 31, 2025. The query and keywords, provided in Supplemental Table 1, included terms such as “ECMO” OR “Extracorporeal membrane oxygenation” OR “Extracorporeal Circulation” and “MANTA” OR “Percutaneous Closure” OR “vascular closure devices” (Supplemental Table 1).

Additionally, the references of the included primary studies and relevant available systematic reviews were screened to search for any further articles of possible interest.

### Selection criteria

Studies that evaluated outcomes associated with the MVCD for decannulating percutaneously placed femoro-femoral VA-ECMO were included. There were no restrictions on publication date or language. Studies were excluded if the VA-ECMO cannulation was performed via open surgical cutdown or if alternative sites (e.g. internal jugular vein or axillary artery) were used. Studies that did not specify the MVCD as the primary closure device, as well as individual case reports, were also excluded. Inclusion criteria targeted original cohort or experimental studies in adults (18+ years).

### Study selection and data extraction

After removing duplicates, two authors (JNC and JRN) independently conducted the study selection process, with any disagreements resolved by a third author (MMV). Initially, studies were screened based on titles and abstracts, with the remaining proceeding to full-text assessment. Attempts were made to contact authors to obtain full-text articles that were not publicly accessible. Two authors (JNC and JRN) performed data extraction from the included studies using a custom-built Microsoft Excel spreadsheet. Data extraction included details on study and patient characteristics, technical specifics, and key outcome measures related to VA-ECMO decannulation using the MVCD.

### Assessment of study quality

Concerning qualitative assessment, the National Heart, Lung, and Blood Institute (NHLBI) Study Quality Assessment Tool was used to assess the risk of bias, adequate reporting, and quality of statistical analysis of observational cohort and case-series studies.^15^ This evaluation was carried out independently by two authors (JNC and JRN). In cases of disagreement, a consensus was reached through discussion, with the assistance of a third author (MMV).

### Outcome measures

This study primarily evaluated technical success rates and complication incidences associated with decannulation using the MVCD in VA-ECMO patients. Key outcomes included rates of open repair following MVCD failure, acute limb ischemia, arterial thrombosis, pseudoaneurysm, hematoma, major bleeding, arterial dissection, arteriovenous fistula, wound infection, and procedure-related mortality.

Technical success was defined as achieving successful hemostasis following VA-ECMO decannulation with the MVCD, without unplanned surgical or endovascular interventions. Bleeding complications were classified according to the Bleeding Academic Research Consortium (BARC) guidelines.^16^ Patients with BARC types 1 or 2 bleeding were classified as having minor bleeding, while those with BARC types 3, 4, or 5 were considered to have major bleeding.

### Quantitative synthesis

A random-effects meta-analysis was conducted using the restricted maximum likelihood method on log-transformed proportions to calculate participants’ pooled incidence of effective and safe percutaneous closure. The pooled estimates and their 95% confidence intervals (95% CI) were back-transformed to their original scale for easier interpretation. Heterogeneity was evaluated through the Q-Cochran *p*-value and the *I*² statistic, with a *p*-value <0.10 and *I*² ⩾50% indicating substantial heterogeneity. Covariates assessed included publication year, participants’ mean age, percentage of male participants, percentage of patients with arterial atherosclerotic risk factors, and percentage of patients using antiplatelet and anticoagulant medications.

All statistical analyses were performed using Open Meta^®^ (MetaMorph, Inc).

## Results

### Search results

**Figure 1.**
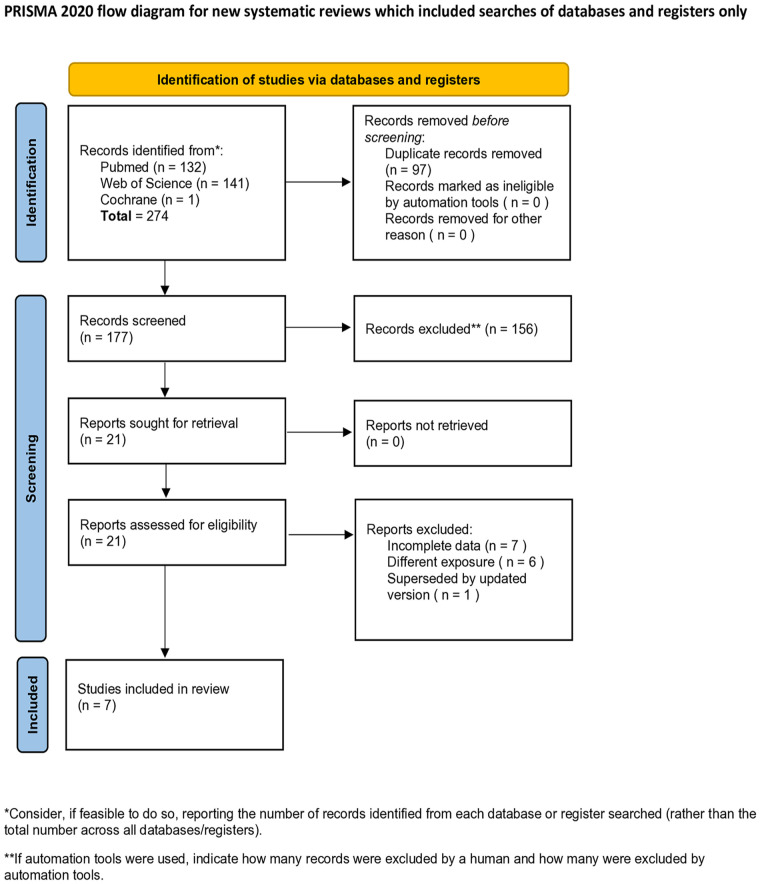
PRISMA flow diagram illustrating the study identification and selection process.

Following the database search and removal of duplicates, a total of 177 studies were screened. After reviewing the titles and abstracts, 156 studies were excluded. Twenty-one studies were deemed eligible for full-text evaluation (Figure 1). The primary reasons for exclusion during the full-text assessment included: incomplete data (*n* = 7), different exposure (*n* = 6), and superseded by updated version (*n* = 1). Seven published articles met the inclusion and exclusion criteria and were incorporated into this study (Table 1).

**Table 1.** Characteristics of the studies included in the systematic review.

Author	Publication year	Journal	Study center	Study design	Study period	Quality of evidence (GRADE)	Number of patients
Bemtgen et al.^17^	2020	*European Heart Journal*	Heart Center Freiburg, Germany	Prospective case series	Jan-June 2019	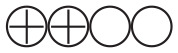 Low	16
Dalén et al.^18^	2022	*Catheterization and Cardiovascular Interventions*	Karolinska University Hospital, Stockholm, Sweden	Retrospective case series	Jan 2018-Oct 2021	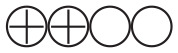 Low	34
Rahman et al.^19^	2023	*SAGE Journals*	Helsinki University Hospital, Finland	Retrospective cohort	Oct 2012-Nov 2020	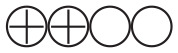 Low	21
Scherer et al.^20^	2023	*Frontiers in Cardiovascular Medicine*	Ludwig-Maximilians-University Munich	Retrospective cohort	Jan 2010-Nov 2021	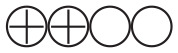 Low	38
Au et al.^21^	2022	*Artificial Organs*	Queen Elizabeth Hospital	Retrospective cohort	Nov 2018-June 2021	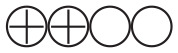 Low	13
Shah et al.^22^	2024	*The Annals of Thoracic Surgery*	University of Maryland School of Medicine, Baltimore	Retrospective cohort	Jan 2018-Jan 2023	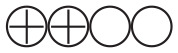 Low	94
Milioglou et al.^23^	2024	*World Journal of Cardiology*	University Hospitals Cleveland Medical Center, Cleveland	Retrospective cohort	Jan 2021-Oct 2023	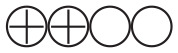 Low	19

### Description of studies

This review includes two observational case series,^17,18^ and five cohort studies.^19–23^ All studies were published recently from 2020 onward. Overall, one of those studies was prospective^17^ and six were retrospective.^18–23^ The included publications were conducted across five countries within three continents: two from North America,^22,23^ four from Europe,^17–20^ and one from Asia.^21^ In total, 235 patients were evaluated across the studies, with sample sizes ranging from 13 to 94 patients per study. The average age of participants was 56.6 years, and 73.61% (*n* = 173) were male (Table 1).

Key characteristics from each study, along with the primary clinical features and comorbidities of the patients, were analyzed (Tables 1 and 2). Procedure-related variables, including VA-ECMO indications, cannula sizes, and decannulation techniques, were also examined (Table 3). Additionally, data on in-hospital outcomes and adverse events associated with cannula removal were compiled, covering surgical open repair, acute limb ischemia, arterial thrombosis, pseudoaneurysm, hematoma, major bleeding, arterial dissection, arteriovenous fistula, wound infection, and procedure-related mortality (Table 4).

**Table 2.** Summary clinical data of included patients.

Author	Bemtgen et al.^17^	Dalén et al.^18^	Rahman et al.^19^	Scherer et al.^20^	Au et al.^21^	Shah et al.^22^	Milioglou et al.^23^
Age (years)	64.7 [59–77.1]	60 ± 13	46 ± 15	57.9 ± 12.0	57.0 [52–65]	54 ± 12	56.8 ± 13.6
Male gender (%)	13 (81.2)	27 (79.4)	17 (80.9)	31 (81.5)	9 (69.2)	64 (68.1)	12 (63.1)
BMI (kg/m^2^)	25.5 [24.2–27.7]	N/A	25.6 ± 5.9	27.9 ± 4.0	N/A	30.1 ± 7.0	29.0 ± 5.4
HT	N/A	N/A	0 (0)	N/A	N/A	54 (57.4)	11 (57.9)
DLD	N/A	N/A	N/A	N/A	N/A	51 (54.2)	N/A
DM	N/A	N/A	0 (0)	N/A	N/A	29 (30.8)	8 (42.1)
Smoking	N/A	N/A	2 (9.5)	N/A	N/A	17 (18.1)	4 (21.0)
CAD	N/A	N/A	4 (19.0)	N/A	N/A	44 (46.8)	15 (78.9)
CKD	N/A	N/A	2 (9.5)	N/A	N/A	14 (14.9)	N/A
AFib	N/A	N/A	1 (4.8)	N/A	N/A	N/A	N/A
Mean PlatBD	65 [61.5–112.3]	154 ± 71	116 ± 82	77.0 [50.0, 108.0]	N/A	N/A	90 [0.9–2.5]
Anticoagulation therapy at cannula removal	yes	yes	No	No	N/A	yes	N/A
Antiplatelet therapy—number of patients	13	29	N/A	34	N/A	N/A	N/A

AFib: atrial fibrillation; BMI: body mass index; CAD: coronary artery disease; CKD: chronic kidney disease; DLD: dyslipidemia; DM: diabetes mellitus; HT: arterial hypertension; N/A: unavailable data; PlatBD: platelet number before decannulation.

Data are *n* (%), mean (SD), median [IQR].

**Table 3.** Procedure related variables.

Author	Indication for VA-ECMO—number of patients (%)	Time spent in ECMO (days)	Procedure setting	Assessment of skin to artery distance	Arterial cannula size	DPC—number of patients (%)	DPC size	Decannulation of DPC	Venous cannula size (Fr)	Decannulation of venous cannula	Follow-up duration (days)
Bemtgen et al.^17^	Cardiac arrest—8 (50) Severe cardiogenic shock—7 (43.8) Fulminant pulmonary embolism—1 (6.2)	3.76 [2.9–6.1]	N/A	No. 5 cm was assumed	15–17 Fr	10 (62.5)	8 Fr	Manual compression	23	Compression with elastic bandages	N/A
Dalén et al.^18^	Acute myocardial infarction—13 (38.2) Postcardiotomy—10 (29.4) Cardiac arrest—5 (14.7) Other—6 (17.6)	6.7 [0.6–18.6]	Catheterization laboratory or operating room	US	17–21 Fr	34 (100)	6–8 Fr	Angio-Seal VCD—22 Manual compression—6 Surgical cutdown—6	21–29	Hemostatic skin sutures and manual compression	53
Rahman et al.^19^	Cardiomyopathy—7 (33.3) Acute coronary syndrome—6 (28.6) Drug toxicity—2 (9.5) Myocarditis—1 (4.8) Other—5 (23.8)	7 ± 7	N/A	US	15–17 Fr	21 (100)	N/A	Angio-Seal VCD	N/A	Skin sutures and compression	N/A
Scherer et al.^20^	STEMI—14 (36.8) Decompensated CMP—12 (31.6) Primary arrhythmia—4 (10.5) NSTEMI—3 (7.9) Myocarditis—2 (5.3) Valvular—1 (2.6) Other—2 (5.3)	4.7 [2.7–7.4]	At bedside	US	15–19 Fr	38 (100)	N/A	Manual compression	22.4	Manual compression	N/A
Au et al.^21^	Acute myocardial infarction—7 (53.8) Shock post open heart operation—4 (30.8) PE—2 (15.4)	5.31 [3.77–6.64]	At bedside	US	15–19 Fr	13 (100)	7 Fr	ProGlide VCD	21–23	Hemostatic skin sutures and compression with femoral compression device	N/A
Shah et al.^22^	Pulmonary embolism—21 (22.3) Postcardiotomy status—9 (9.6) End-stage heart failure—16 (17.0) Acute coronary syndrome—24 (25.5) Other—24 (25.5)	6 [4–11]	Operation room and bedside	CT and US	<17 Fr—1 (1.1) 17–18 Fr—57 (60.6) 19–20 Fr—34 (36.1) >20 Fr—2 (2.1)	N/A	6 Fr >6 Fr	Angio-Seal VCD, MYNX VCD or manual compression.	N/A	Pursestring suture with percutaneous removal	161 [74–387]
Milioglou et al.^23^	Acute coronary syndrome—7 (36.8) Cardiac arrest—4 (21.0) Cardiogenic shock—4 (21.0) Postcardiotomy—2 (10.5) TAVI—2 (10.5)	5.00 [2–20]	Operation room and bedside	No. 8 cm was assumed	17 Fr—9 (47.4) 18 Fr—1 (5.3) 19 Fr—8 (42.1) 21 Fr—1 (5.3)	19 (100)	N/A	N/A	N/A	N/A	N/A

DPC: distal perfusion catheter; ECMO: extracorporeal membrane oxygenation; LVAD: left ventricular assist device; N/A: unavailable data; NSTEMI: non-ST-elevation myocardial infarction; PE: pulmonary embolism; STEMI: ST-elevation myocardial infarction; VCD: vascular closure device.

Data are *n* (%), median [IQR].

**Table 4.** Short-term outcomes and adverse events.

Author	Bemtgen et al.^17^	Dalén et al.^18^	Rahman et al.^19^	Scherer et al.^20^	Au et al.^21^	Shah et al.^22^	Milioglou et al.^23^
Manta efficacy (%)	16 (100)	28 (82.3)	20 (95.2)	37 (97.4)	13 (100)	85 (90.4)	19 (100)
Open repair following Manta VCD failure (%)	0 (0)	6 (17.6)	0 (0)	1 (2.6)	0 (0)	4 (4.2)	0
Acute limb ischemia (%)	0 (0)	3 (8.8)	0 (0)	2 (5.3)	1 (7.7)	5 (5.3)	2 (10.5)
Arterial thrombosis (%)	3 (18.8)	3 (8.8)	0 (0)	2 (5.3)	0 (0)	12 (12.8)	N/A
Pseudoaneurysm (%)	4 (25)	1 (2.9)	0 (0)	1 (2.6)	0 (0)	3 (3.2)	N/A
Hematoma (%)	N/A	1 (2.9)	2 (9.5)	N/A	N/A	8 (8.5)	N/A
Major bleeding (BARC >3) (%)	0 (0)	1 (2.9)	4 (19.0)	1 (2.6)	0 (0)	5 (5.3)	1 (5.2)
Arterial dissection (%)	N/A	N/A	N/A	0 (0)	1 (7.7)	3 (3.2)	N/A
Arteriovenous fistula (%)	N/A	N/A	0 (0)	N/A	0 (0)	0 (0)	N/A
Wound infection (%)	0 (0)	0 (0)	0 (0)	N/A	0 (0)	7 (7.4)	0 (0)
Procedure related death (%)	0 (0)	0 (0)	0 (0)	0 (0)	0 (0)	0 (0)	0 (0)

BARC: Bleeding Academic Research Consortium; Manta VCD: Manta vascular closure device; N/A: unavailable data.

### Studies quality

**Figure 2.**
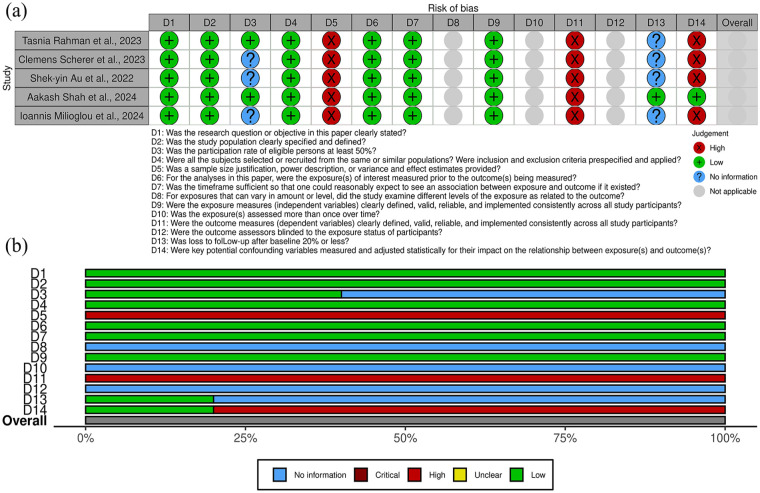
(a) Risk of bias assessment for all cohort studies included in the systematic review, presented by individual article and (b) risk of bias assessment for all included cohort studies, presented by individual item.

**Figure 3.**
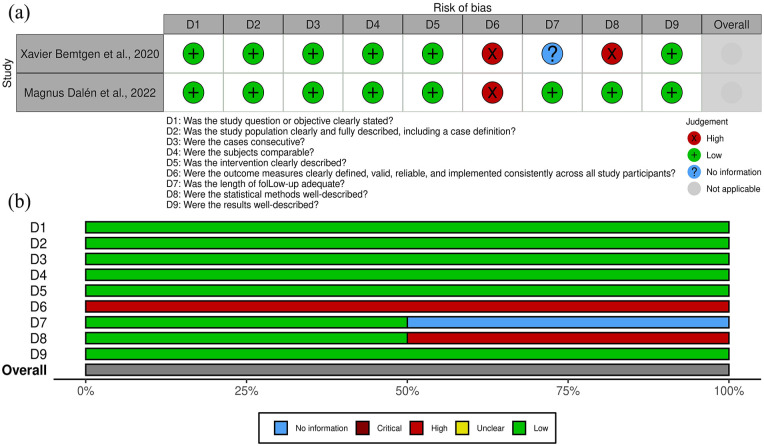
(a) Risk of bias assessment for all case series studies included in the systematic review, organized by article and (b) risk of bias assessment for all included case series studies, presented by specific item.

Figures 2 and 3 outline the risk of bias in the included studies. Figure 2(a) details individual assessments for each observational cohort, while Figure 3(a) summarizes key areas of bias across cohorts.

In the case series, the domain with the highest risk of bias was the definition of outcomes (question D6). This reflects inconsistencies or a lack of clarity in how outcomes were specified or measured across the studies.

For cohort studies, the primary sources of bias were related to the determination of sample size adequacy (question D5), the definition of outcomes (question D11), and the consideration of confounding factors in the analysis (question D14). These issues highlight limitations in study design, including insufficient statistical power, vague or incomplete outcome definitions, and inadequate adjustment for potential biases in the analyses.

There were no prohibitive concerns that would compromise the reliability or applicability of the results.

### Decannulation techniques

All included studies had reported their technique for decannulation of the arterial cannula. VA– ECMO decannulation was performed either in the operating room,^18,22,23^ in the catheterization laboratory^18^ or at bedside.^20–23^

The sizes of the arterial cannulas used for VA–ECMO ranged from 15 to 21 F or more. Of the 235 patients, only one had the depth locator used during decannulation, as originally described in the previous study by Shah et al.,^24^ as emergent VA-ECMO cannulations generally did not allow for its use at the time of cannula placement. In four studies, the distance between the skin and the artery was determined by ultrasound examination prior to VA–ECMO decannulation^18–21^ while one study relied on measurements derived from both computed tomography scan and ultrasound.^22^ In two other study, the distance between the skin and the artery was not determined and was assumed to be five^17^ or eight^23^ centimeters. In preparation for MVCD placement during ECMO decannulation, wire access to the arterial cannula was achieved using several techniques. Most studies described clamping and separating the cannula from the ECMO circuit, followed by guidewire insertion under sterile conditions.^17,18,21,23^ Another method introduced a stiff guidewire (Amplatz Extra Stiff) with an introducer dilator, confirmed via imaging (echocardiography or fluoroscopy) in the descending aorta.^19^ Additionally, a J-tip guidewire was used, with some protocols employing a hemostasis valve Y connector to facilitate controlled insertion for MANTA^®^ VCD deployment.^20^

The size of the venous cannulas used for VA–ECMO ranged from 21 to 29 F.^17,18,20,21^ Following decannulation of the venous access site during ECMO weaning, various techniques were used to achieve hemostasis. Some studies reported applying compression with elastic bandages^17^ or using a figure-of-eight skin suture followed by manual pressure for 10 min.^18^ Other techniques included placing a Prolene purse-string suture around the cannula insertion site,^22^ or a deep Z-shaped suture with a sandbag applied for 4 h to control bleeding.^19^ Additional approaches involved continued manual compression for at least 5 min with a pressure bandage applied for 12 h,^20^ or using a C-clamp for 30 min on both arterial and venous wounds.^21^ No studies mentioned the use of a VCD for the decannulation of a venous cannula.

The size of the distal perfusion catheter used for VA–ECMO ranged from 6 to 8 F.^17,18,21,22^ Following ECMO decannulation, several methods were used to remove and close the distal perfusion catheter access site. Techniques included the use of other closure devices in the superficial femoral artery (SFA),^18,19,21,22^ while some approaches relied on manual compression^17,18,20^ or surgical cutdown^18^ based on surgeon’s discretion.^18^

### Main findings and meta-analysis

**Figure 4.**
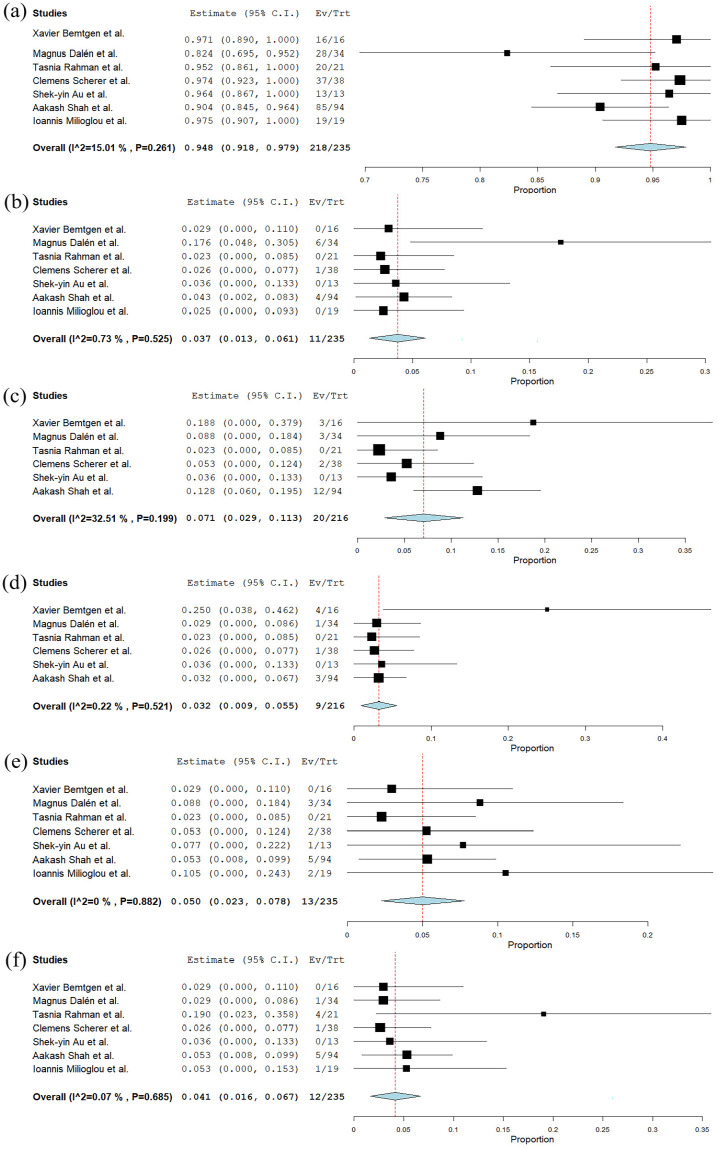
(a) Forest plot illustrating the technical success rate of the MANTA vascular closure device in VA-ECMO decannulation across the included studies, (b) forest plot illustrating the incidence of emergency open repair cases following the use of the MANTA vascular closure device in VA-ECMO decannulation across the included studies, (c) forest plot showing the incidence of arterial thrombosis associated with the use of the MANTA vascular closure device in VA-ECMO decannulation across the included studies, (d) forest plot showing the incidence of pseudoaneurysms associated with the use of the MANTA vascular closure device in VA-ECMO decannulation across the included studies, (e) forest plot showing the incidence of acute limb ischemia associated with the use of the MANTA vascular closure device in VA-ECMO decannulation across the included studies, and (f) forest plot showing the incidence of major bleeding associated with the use of the MANTA vascular closure device in VA-ECMO decannulation across the included studies.

In 235 patients, the meta-analytical technical success rate of MVCD in VA-ECMO decannulation was 94.8% (95% CI 91.8%–97.9%, Standard error (SE) 1.6%, *p* < 0.001) *I*^2^ = 15.01% (Fixed effects).^17–23^ In all results of leave-one-out sensitivity, the overall estimate remained stable across all iterations. VCD failure was defined as failure of MANTA^®^ to achieve hemostasis at the arteriotomy site, requiring alternative treatment to manual compression. Of the 17 patients with MANTA^®^ device failure, 11 were managed primarily through surgical cutdown.^18,20,22^ One failure case was successfully treated using endovascular ballooning,^19^ three cases were resolved with thrombectomy^22^ and two other failure cases were managed with endovascular interventions.^22^ The incidence of reported emergency open repair following the failure of the MVCD device was 3.7% (95% CI 1.3%–6.1%, Standard error (SE) 1.2%, *p* = 0.002) *I*^2^ = 72.8% (mixed effects; Figure 4, Table 4).^17–23^

The rate of patients experiencing arterial thrombosis was 7.1% (95% CI 2.9%–11.3%, Standard error (SE) 0.21%, *p* < 0.001) *I*^2^ = 32.5% (fixed effects; Figure 4, Table 4).^17–22^ Among the 20 cases of thrombosis,^17,18,20,22^ only two cases required surgical cutdown, involving MANTA^®^ plug removal, catheter thrombectomy, and patch reconstruction of the CFA.^18,20^ The remaining cases were managed with thrombectomy^22^ and conservative treatment.^17,20,22^

The incidence of patients with pseudoaneurysm after decannulation was 3.2% (95% CI 0.9%–5.5%, Standard error (SE) 1.2%, *p* = 0.007) *I*^2^ = 0.22% (fixed effects; Figure 4, Table 4).^17–22^ Five pseudoaneurysms were managed conservatively,^17,22^ while three received percutaneous thrombin injections.^17,20^ The remaining pseudoaneurysm was located at the CFA, requiring surgical cutdown with CFA patch repair.^18^

The incidence of acute limb ischemia after VA-ECMO decannulation was 5.0% (95% CI 2.3%–7.8%, Standard error (SE) 1.4%, *p* < 0.001) *I*^2^ = 0% (fixed effects; Figure 4, Table 4).^17–23^ Four cases required surgical cutdown with additional catheter thrombectomy or CFA patch reconstruction.^18,20^ Four cases required endovascular interventions^22,23^ and the remaining cases were conservatively.^20–22^

The observed rate of major arterial bleeding was 4.1% (95% CI 1.6%–6.7%, Standard error (SE) 1.3%, *p* < 0.001) *I*^2^ = 22.4%.^17–23^ All cases were managed conservatively,^19,22,23^ except for one treated with endovascular ballooning,^19^ and another requiring surgical cutdown (Figure 4).^18^

In 197 patients, no study reported any case of wound infection.^17–19,21–23^ Out of 107 patients, there were only 4 reports of arterial dissections.^21,22^ Across 149 patients, the occurrence of hematoma varied from 2.94% to 9.5%.^18,19,22^ Among 128 patients, no study reported any case of arterio-venous fistula. Concerning other short-term outcomes in patients that underwent percutaneous VA-ECMO decannulation, available data was sparse but further withdrawn and displayed in Table 4.

## Discussion

This systematic review and meta-analysis investigate the use of the MVCD in VA-ECMO decannulation, offering important insights into its effectiveness for managing large-bore vascular access. It presents evidence indicating that this method is associated with a high technical success rate and a low incidence of complications related to decannulation.

Several studies have examined the MVCD’s safety and effectiveness in closing large-bore arteriotomies. For instance, the SAFE MANTA clinical trial reported a technical success rate of 97.7% in patients undergoing percutaneous TAVI and T/EVAR, highlighting MANTA^®^’s strong performance and reliability in these procedures.^9^ While MANTA^®^ has shown promising outcomes in large-bore arterial access closure, managing access sites after VA-ECMO presents additional challenges. These arise from the extended support duration and the ECMO-associated coagulopathy that can complicate hemostasis.^25^ Despite these challenges, a technical success rate of 94.8% was detected in this systematic review, consistent with previous findings.

Due to its minimally invasive nature, VA-ECMO decannulation with the MVCD has demonstrated a favorable composite outcome regarding vascular complications and wound infections compared to surgical decannulation.^9^ This approach avoids the need for transport to the operating room and general anesthesia, as well as a groin incision—an area prone to infection, poor healing, and lymphatic leakage.^26^ Supporting this, the study by Rahman et al. reported that decannulation with the MVCD resulted in significantly lower rates of hematomas, seromas, and surgical site infections requiring intervention compared to surgical decannulation.^19^

In addition to the MVCD, the suture-based ProGlide VCD (Abbott, Chicago, IL, USA) is another option for percutaneous decannulation in VA-ECMO patients.^6,27^ Although suture-based closure devices have been used for this purpose, they have yet to become widely adopted due to their requirement for pre-closure before cannulation, which may delay ECMO initiation in rapidly deteriorating patients. Additionally, the exposed sutures between insertion and decannulation could increase the risk of wound infection.^28^ The systematic review included two studies comparing the MANTA^®^ and ProGlide VCDs for VA-ECMO decannulation. Both reported higher success rates and fewer complications with the MVCD group.^20,21,27^ Despite these findings, the choice of device is only sometimes straightforward. Several clinical factors guide the decision for the best decannulation approach. For example, if infection is present or suspected, the ProGlide system may be preferred over the MANTA^®^ due to the infection risk associated with the MANTA^®^’s collagen plug. Similarly, if there is a likelihood of needing to re-establish vascular access on the same side, ProGlide might be advantageous since MANTA^®^’s components take about 6 months to reabsorb.^8^ In selected scenarios, particularly in patients with a narrower native common femoral artery, the elevation of the toggle on the MANTA^®^ VCD can result in artery occlusion. In these instances, the ProGlide VCD may be a better option, as it requires a minimum vessel size of 5 mm, smaller than the 6 mm requirement for the MANTA^®^ device.^11^

In this review, the depth locator was used in only one patient, due to the emergency nature of VA-ECMO cannulations.^24^ CT scans^22^ and ultrasounds,^17–22^ depending on the preference of the authors, were used before decannulation to verify depth measurements, maximizing accuracy.

In the study by Dalén et al. Duplex scan after decannulation was used solely to confirm distal perfusion, without visualizing the MANTA^®^ device position.^18^ This approach may miss suboptimal device positioning, potentially explaining three cases of intravascular MANTA^®^ placement that resulted in two thromboses and one pseudoaneurysm requiring surgical intervention. Intravascular positioning can occur if the toggle encounters arterial calcification or a branch, pushing the collagen plug inward. Ultrasound guidance can mitigate this risk by allowing precise release of the toggle near the arterial wall.^11,29^ To prevent these complications, the deployment of the MANTA^®^ device should be performed under simultaneous ultrasound guidance, a technique recently described in several studies with MANTA^®^ in transcatheter aortic valve implantation (TAVI),^30^ and in VA-ECMO decannulation.^31^

Prolonged ECMO cannulation in the femoral artery can lead to fibrotic arteriotomy edges, complicating effective closure. The MANTA^®^ device, typically effective for arteriotomies with flexible edges after shorter procedures, may struggle with fibrotic openings. Dalén et al. described a case where an 11.5-day ECMO duration resulted in rigid, fibrotic edges, causing the MANTA^®^ device to inadequately seal the vessel completely.^18^

It is worth nothing that the use of anticoagulation in patients undergoing VA-ECMO is common and is done to diminish circuit-associated thrombotic risks^32^; additionally, VA-ECMO is known to provoke an acquired von Willebrand disease.^33^ However, this practice may potentially lead to increased rates of hemorrhagic complications. Only two studies reported stopping anticoagulation before VA-ECMO decannulation (1 and 4 h before).^19,20^

In most studies, limb ischemia was evaluated through Doppler ultrasound, having just one report^17^ of use of Near-infrared spectroscopy (NIRS) for monitoring limb perfusion, an important tool in VA-ECMO decannulation.^34^ No data about the selection of limb side was reported either.

Although some deaths have been reported,^18,20,22,23^ none were attributed to VA-ECMO decannulation or the use of the MANTA^®^ device. Therefore, no procedure-related deaths were observed, highlighting the strong safety profile of this technique.

This study encountered several limitations. The systematic review included a limited number of eligible articles, with small sample sizes without justification or power analysis, resulting in low precision and potentially compromising external validity. Notably, no randomized clinical trials were identified in the literature. The included studies lacked randomization, with treatment decisions likely influenced by surgeon’s preference, experience, and patient selection, which may have impacted the reported success rates of the MVCD. Significant heterogeneity was observed across studies, particularly regarding baseline patient characteristics, study designs, and methodologies, reflecting diverse indications for VA-ECMO cannulation. Furthermore, inconsistent or insufficient follow-up periods hindered the ability to accurately assess outcome rates at 6 months, especially for vascular complications. Additionally, the studies were susceptible to publication bias, which likely limited heterogeneity in the meta-analyses and may have underestimated the complication rates associated with VA-ECMO decannulation using the MVCD.

## Conclusion

This review suggests that MVCD is a promising, effective and reliable approach for achieving hemostasis following percutaneous VA-ECMO decannulation, with an acceptable success rate, and minimal major complications reported. It contributes to a growing body of evidence supporting the potential role of MVCD as a minimally invasive option for complex VA-ECMO decannulation procedures. Larger, multicenter studies with uniform follow-up are needed to confirm these findings and precisely assess complication rates.

## Supplemental Material

sj-pdf-1-jva-10.1177_11297298251325391 – Supplemental material for Efficacy, safety, and complications of manta vascular closure device in VA-ECMO decannulation: A systematic review and meta-analysis Supplemental material, sj-pdf-1-jva-10.1177_11297298251325391 for Efficacy, safety, and complications of manta vascular closure device in VA-ECMO decannulation: A systematic review and meta-analysis by Joana Nunes-Carvalho, Eduardo Silva, Paolo Spath, Leonardo Araújo-Andrade, Nicola Troisi and João Rocha Neves in The Journal of Vascular Access
