# Supplementary material for: Efficacy, safety, and complications of manta vascular closure device in VA-ECMO decannulation: A systematic review and meta-analysis
Source: J Vasc Access. 2025 Mar 21;27(1):39–51. doi: 10.1177/11297298251325391 (PMC12812181; doi:10.1177/11297298251325391)
Supplement: sj-pdf-1-jva-10.1177_11297298251325391 – Supplemental material for Efficacy, safety, and complications of manta vascular closure device in VA-ECMO decannulation: A systematic review and meta-analysis [file sj-pdf-1-jva-10.1177_11297298251325391.pdf]

Supplemental Table I: Search Strategy

Databases	Query and keywords	Articles found
**Pubmed**	(“ECMO” OR “Extracorporeal membrane oxygenation” OR “Extracorporeal Circulation” OR “ECLS” OR “extracoporeal life-support” OR “venous-arterial” OR “extracorporeal cardiopulmonary resuscitation” OR “ECPR” OR “venoarterial” OR “Mechanical circulatory support”) AND ("Manta” OR “MANTA Closure” OR "vascular closure devices" OR ("vascular" AND "closure" AND "device") OR "vascular closure device" OR “Vascular closure” OR “Closure device” OR “Percutaneous closure” OR “artery closure” OR “Arterial Puncture closure”)	132
**Web of Science**	(“ECMO” OR “Extracorporeal membrane oxygenation” OR “Extracorporeal Circulation” OR “ECLS” OR “extracoporeal life-support” OR “venous-arterial” OR “extracorporeal cardiopulmonary resuscitation” OR “ECPR” OR “venoarterial” OR “Mechanical circulatory support”) AND ("Manta” OR “MANTA Closure” OR "vascular closure devices" OR ("vascular" AND "closure" AND "device") OR "vascular closure device" OR “Vascular closure” OR “Closure device” OR “Percutaneous closure” OR “artery closure” OR “Arterial Puncture closure”)	141
**Cochrane**	(“ECMO” OR “Extracorporeal membrane oxygenation” OR “Extracorporeal Circulation” OR “ECLS” OR “extracoporeal life-support” OR “venous-arterial” OR “extracorporeal cardiopulmonary resuscitation” OR “ECPR” OR “venoarterial” OR “Mechanical circulatory support”) AND	1

("Manta” OR “MANTA Closure” OR "vascular closure devices" OR ("vascular" AND "closure" AND "device") OR "vascular closure device" OR “Vascular closure” OR “Closure device” OR “Percutaneous closure” OR “artery closure” OR “Arterial Puncture closure”)
